# COVID-19-Related Spontaneous Vertebral Artery Dissection: A Case Report

**DOI:** 10.7759/cureus.84265

**Published:** 2025-05-17

**Authors:** Thamer AlAifan, Aisha Halawani, Nora Shalabi, Abdulmalek M Yaghmour, Abdulrazak M Sakhakhni, Wasan S Alzahrani, Rawnaa N Haqqi

**Affiliations:** 1 Critical Care Medicine, King Abdulaziz Medical City in Jeddah, Jeddah, SAU; 2 Research Office, King Abdullah International Medical Research Center, Jeddah, SAU; 3 Medicine, King Saud Bin Abdulaziz University for Health Sciences, Jeddah, SAU; 4 Neuroradiology, King Abdulaziz Medical City in Jeddah, Jeddah, SAU

**Keywords:** case report, covid 19, neurology and critical care, neurovascular disease, spontaneous vertebral artery dissection

## Abstract

Coronavirus disease 2019 (COVID-19), caused by severe acute respiratory syndrome coronavirus 2 (SARS-CoV-2), is associated with various vascular complications, including arterial dissections. This case report examines a potential link between COVID-19 and spontaneous vertebral artery dissection (VAD) in a 48-year-old female with hypertension, uncontrolled diabetes mellitus, heart failure with reduced ejection fraction, end-stage renal disease requiring hemodialysis, and obesity. She presented with a severe headache, acute confusion, and left-sided weakness, testing positive for COVID-19 via polymerase chain reaction. Neurological deficits were evident, and imaging confirmed a left vertebral artery dissection, resulting in cerebellar infarctions and obstructive hydrocephalus, ultimately leading to brain death. This case suggests that severe COVID-19 may precipitate VAD, particularly in patients with significant comorbidities. Potential mechanisms include endothelial dysfunction, inflammation, and hypercoagulability. Management typically involves anticoagulation or antiplatelet therapy, with surgical intervention in refractory cases. This case underscores the need for heightened awareness of vascular complications in COVID-19 patients with comorbidities. Further research is essential to elucidate the interactions between COVID-19 and chronic conditions contributing to severe systemic complications.

## Introduction

COVID-19, caused by severe acute respiratory syndrome coronavirus 2 (SARS-CoV-2), was first detected in late 2019 and quickly spread via respiratory droplets [1]. The disease presents with symptoms ranging from mild to severe, potentially leading to death in extreme cases [1]. Notably, multiple cases have been reported linking arterial dissections to SARS-CoV-2 infection [2]. Vascular dissections are characterized by a tear in the arterial wall, leading to the separation of the vessel’s layers and the formation of a hematoma [3]. Vertebral artery dissection (VAD), involving one or both vertebral arteries, results in various neurological deficits depending on the dissection’s location, type, extent of blockage, and status of other supplying arteries [3]. Epidemiologically, the estimated incidence of spontaneous vertebral artery dissection is 1 to 1.5 per 100,000 [4]. Driven by several critical mechanisms, SARS-CoV-2 interacts with angiotensin-converting enzyme 2 (ACE2) receptors on endothelial cells, causing dysfunction and inflammation that may contribute to arterial dissection [5,6]. Additionally, the use of high-dose corticosteroids in COVID-19 patients can further compromise arterial walls, increasing the risk of spontaneous dissections [7]. The interplay of these factors, endothelial damage, inflammation, increased clotting tendency, and corticosteroid use, significantly contributes to the elevated incidence of arterial dissections observed in individuals with COVID-19. Similarly, messenger ribonucleic acid (mRNA) vaccines may increase the risk of arterial dissections by activating systemic inflammation and proinflammatory pathways, potentially leading to immune-mediated responses or exacerbating pre-existing conditions [8]. In conclusion, COVID-19 may elevate the risk of arterial dissections; however, direct evidence is currently lacking, underscoring the need for further research to better understand and mitigate these risks. The aim of presenting this case is to present a case report of spontaneous VAD associated with COVID-19, particularly those with multiple comorbidities, and to review similar cases from the literature. Reporting such cases helps assess the impact of COVID-19, which remains a global pandemic.

## Case presentation

A 48-year-old, non-smoking female presented to the hospital emergency department with a severe headache and acute confusion. Her past medical history included hypertension, uncontrolled diabetes mellitus, heart failure with reduced ejection fraction (HFrEF) of 49%, end-stage renal disease (ESRD) on hemodialysis with frequent missed sessions, and obesity (body mass index (BMI), 84.79 kg/m²). Upon admission, she exhibited left-sided weakness that had begun four days earlier, along with acute confusion, occipital headache, and neck pain. She had missed her last hemodialysis session. There was no reported loss of consciousness, seizure activity, choking attacks, or slurred speech. The patient also denied chest pain, syncope, palpitations, or any history of fever, vomiting, falls, trauma, photophobia, blurry vision, or previous similar episodes. Her family and psychosocial histories were unremarkable.

On physical examination, the patient’s Glasgow Coma Scale (GCS) score was 15/15 upon arrival, with vital signs as follows: blood pressure 229/98 mmHg, heart rate 87 beats per minute (bpm), oxygen saturation 99% on room air, respiratory rate 19 breaths/min, and temperature 37°C. She had bilaterally equal and reactive pupils, a left-sided lower motor neuron facial palsy, and loss of the left nasolabial fold. Muscle power examination revealed right upper limb (4/5), right lower limb (3/5), left upper limb (2/5), and left lower limb (2/5), with brisk reflexes bilaterally. Sensory examination indicated reduced pinprick and temperature sensation on the left side in both upper and lower limbs compared to the right, along with decreased proprioception bilaterally in both toes. Lung auscultation revealed bilateral basal crepitations. Examination of the lower extremities showed bilateral diabetic foot changes, including amputated toes, a Charcot joint, and multiple ulcers. The remainder of the physical examination was unremarkable. Initial laboratory tests are shown in Table 1.

**Table 1:** Laboratory tests at presentation

Parameter	Patient Value	Reference Range
Hemoglobin	9.5 g/dL	11.5-16.5 g/dL
White blood cell (WBC) count	7.9 x 10^9/L	4.0-11.0 x 10^9/L
Partial thromboplastin time	28	25-35
International normalized ratio	1.1	>1.5
High-sensitivity troponin (Hs-TnI)	165.7 pg/mL	1.9-34.2 pg/mL
Blood glucose	4 mmol/L	2.9-7.8 mmol/L
Sodium	133 mmol/L	135-145 mmol/L
Potassium	5.5 mmol/L	3.5-5 mmol/L

A COVID-19 polymerase chain reaction (PCR) nasopharyngeal swab, performed per hospital policy, returned positive. The patient’s vaccination status was unknown. An urgent brain computed tomography (CT) scan showed extensive hypodensity involving the left cerebellar hemisphere, suggestive of an acute or subacute posterior inferior cerebellar artery (PICA) infarction. The patient was admitted as a case of PICA stroke for further workup, hypertension management, and nephrology follow-up.

**Figure 1:**
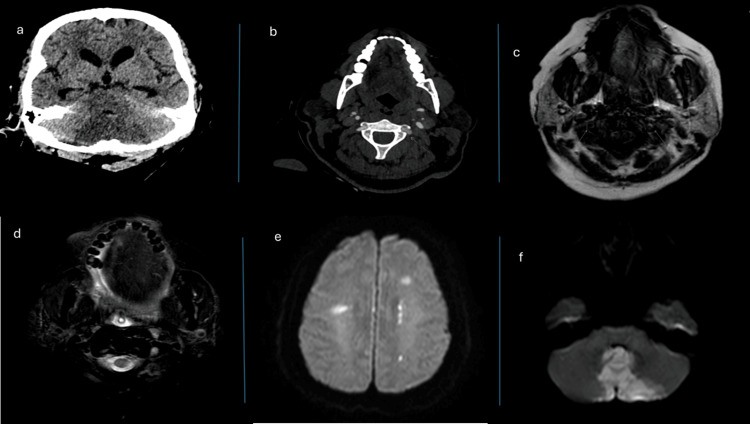
CT carotid angiogram and four MRI of a patient with spontaneous vertebral artery dissection (a) Unenhanced CT scan at the time of presentation demonstrating cerebellar hypodensity along the posterior superior cerebellar artery. (b) Faint opacification of the left vertebral artery with subtle linear filling defect. (c) and (d) Axial T1 and T2 demonstrate loss of normal signal void at the left vertebral artery on T2 with high T1 signal abnormality consistent with vertebral artery dissection. (e) and (f) Corresponding diffusion-weighted images shows showering emboli and multiple areas of restricted diffusion at the cerebral white matter with cerebellar infarcts along the distribution of posterior inferior cerebellar artery and perforators.

To further evaluate her condition, brain magnetic resonance imaging (MRI) and carotid CT angiography were ordered. Carotid CT angiography, performed 14 hours post-admission, demonstrated bilateral cerebellar infarcts causing mild hydrocephalus and non-opacified PICA bilaterally, including their branches. A faint opacification of the left vertebral artery with a linear filling defect suggested dissection (Figure 1).

Atherosclerotic changes were noted in the cavernous part of the temporal carotid artery and thoracic aorta. CT pulmonary angiography showed no evidence of pulmonary embolism. The patient received aspirin, and her blood pressure was initially controlled with nifedipine and hydralazine.

After 24 hours, the patient suddenly became unconscious, necessitating intubation and stabilization. Brain MRI revealed acute infarction involving the bilateral inferior cerebellar hemispheres and the left hemi-medulla oblongata in the PICA territory, along with bilateral scattered small acute microinfarcts affecting the superior vermis, left lateral upper pons, corona radiata, centrum semiovale of both cerebral hemispheres, and the splenium of the corpus callosum. Notably, an acute dissection of the left vertebral artery caused a mass effect on the fourth ventricle, with upstream ventricular dilatation and mild transependymal permeation indicative of acute obstructive hydrocephalus (Figure 1).

Over the next 24 hours, the patient developed anisocoria (left pupil 3 mm, right pupil 5 mm). An external ventricular drain was inserted, but her condition did not improve. A subsequent CT scan showed diffuse loss of gray-white matter differentiation in the bilateral cerebral hemispheres, hyperdense falx cerebri and tentorium cerebelli, and tonsillar herniation. She was later diagnosed with brain death based on clinical criteria and absent cerebral blood flow on a nuclear scan.

## Discussion

In this report, we highlight a unique interplay between severe COVID-19 infection, among other risk factors, and vascular structures leading to vertebral artery dissection (VAD). We describe a 48-year-old female patient with a history of hypertension, uncontrolled diabetes mellitus, ESRD, and obesity who presented to the emergency department with acute neurological symptoms and a PCR-confirmed COVID-19 infection. Imaging revealed bilateral cerebellar infarcts and an acute left VAD, ultimately resulting in obstructive hydrocephalus, brainstem compromise, and brain death. We speculate that the patient’s unfortunate outcome resulted from the combined effects of her multiple comorbidities and COVID-19 infection; however, this temporal association with COVID-19 may not establish causality, and the precise mechanisms linking SARS-CoV-2 to ischemic events and vascular complications remain unclear.

Spontaneous vertebral artery dissection (sVAD) is a rare condition characterized by the separation of arterial wall layers, leading to blood accumulation within the vessel layers and potentially causing stenosis, thrombus formation, or embolism. Although the exact triggers are unclear, it is believed that underlying structural weaknesses, genetic predispositions, and minor trauma may contribute to its occurrence [9]. In clinical settings, patients with sVAD typically present with symptoms such as neck pain, headache, or neurological deficits [10,11]. While spontaneous VAD has been reported in non-COVID contexts, its occurrence in COVID-19 patients suggests a potential direct or indirect causative relationship, possibly related to systemic inflammation, endothelial dysfunction, and hypercoagulable states.

The pathophysiology linking SARS-CoV-2 to vascular complications is multifaceted. COVID-19-associated coagulopathy, characterized by elevated D-dimer levels, prolonged prothrombin time, thrombocytopenia, and increased inflammatory markers, is a key contributor to these complications [12,13]. Additionally, the interaction between SARS-CoV-2 and the angiotensin-converting enzyme 2 (ACE2) receptor significantly contributes to vascular dysfunction. The virus binds to ACE2, widely expressed in vascular endothelial cells, leading to its downregulation. This results in an imbalance between the classical and alternative renin-angiotensin system (RAS) pathways [14]. Upregulation of the ACE-Ang II-AT1R axis promotes vasoconstriction, oxidative stress, and inflammation, while downregulation of the ACE2-Ang-(1-7)-Mas axis reduces protective responses such as vasodilation and antithrombotic activity [15,16]. Furthermore, the hyperinflammatory response in severe COVID-19 cases, often termed a "cytokine storm," exacerbates vascular instability. Elevated levels of interleukin-6 (IL-6), tumor necrosis factor-alpha (TNF-α), and C-reactive protein (CRP) are associated with hypercoagulable states, promoting thrombus formation and vascular injury [17].

Management of spontaneous vertebral artery dissection (sVAD) typically begins with diagnostic imaging, such as CT angiography (CTA) or MRI, to confirm the dissection and assess its extent [18]. The primary therapeutic goal is to prevent stroke and thromboembolic events through anticoagulation or antiplatelet therapy [18,19]. Medications such as beta-blockers or calcium channel blockers are also used to control blood pressure and minimize stress on the vascular wall [18]. If pharmacological treatment is insufficient, surgical or endovascular interventions may be necessary to stabilize the dissection and prevent further complications [18,19].

## Conclusions

COVID-19 was and remains an active worldwide issue, with its full scope and challenges yet to be fully uncovered. This case report of spontaneous vertebral artery dissection (sVAD) in a patient with multiple comorbidities shows a rare but severe complication. The interaction between SARS-CoV-2 and chronic diseases, such as hypertension and diabetes, increases the likelihood of adverse vascular events, potentially driven by mechanisms like ACE2 receptor interaction, endothelial dysfunction, and hyperinflammation. Reporting such cases is essential, as it adds to the current literature and enhances our perception of the disease’s diverse manifestations. However, as a case report, its generalizability is limited, necessitating further research with larger cohorts to better understand the potential for severe vascular complications in COVID-19 patients.
